# Lateral line morphology, sensory perception and collective behaviour in African cichlid fish

**DOI:** 10.1098/rsos.221478

**Published:** 2023-01-25

**Authors:** Elliott Scott, Duncan E. Edgley, Alan Smith, Domino A. Joyce, Martin J. Genner, Christos C. Ioannou, Sabine Hauert

**Affiliations:** ^1^ Department of Engineering Mathematics, University of Bristol, Bristol BS8 1UB, UK; ^2^ School of Biological Sciences, University of Bristol, Bristol BS8 1TQ, UK; ^3^ Department of Biological and Marine Sciences, University of Hull, Hull HU6 7RX, UK

**Keywords:** lateral line, collective behaviour, bioinspiration, sensors, cichlids

## Abstract

The lateral line system of fishes provides cues for collective behaviour, such as shoaling, but it remains unclear how anatomical lateral line variation leads to behavioural differences among species. Here we studied associations between lateral line morphology and collective behaviour using two morphologically divergent species and their second-generation hybrids. We identify collective behaviours associated with variation in canal and superficial lateral line morphology, with closer proximities to neighbouring fish associated with larger canal pore sizes and fewer superficial neuromasts. A mechanistic understanding of the observed associations was provided by hydrodynamic modelling of an artificial lateral line sensor, which showed that simulated canal-based neuromasts were less susceptible to saturation during unidirectional movement than simulated superficial neuromasts, while increasing the canal pore size of the simulated lateral line sensor elevated sensitivity to vortices shed by neighbouring fish. Our results propose a mechanism behind lateral line flow sensing during collective behaviour in fishes.

## 1. Introduction

**Figure 1.**
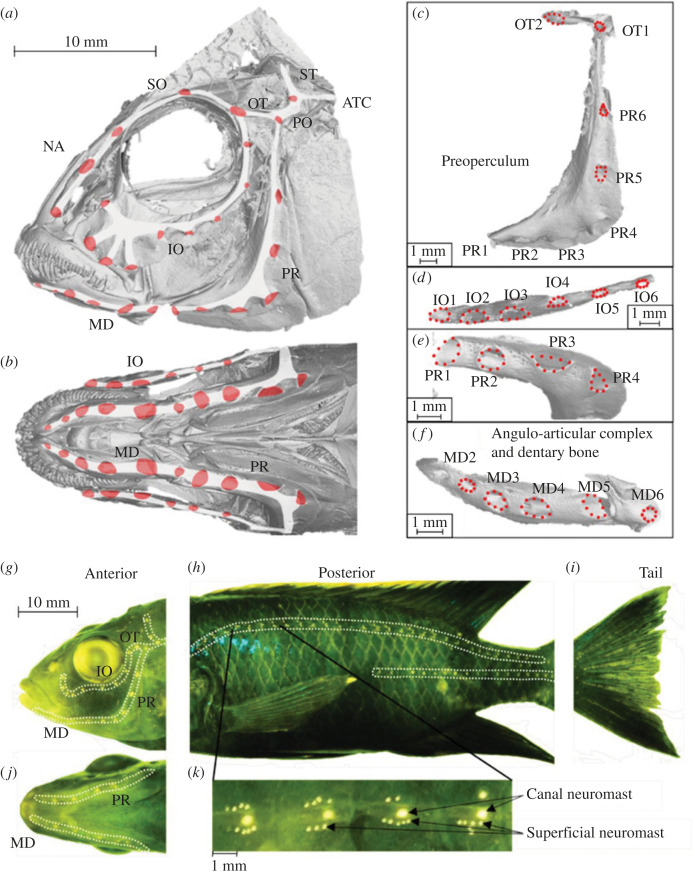
Morphology quantified in this study in an example from the hybrids group. (*a*) Lateral view CT scans showing anterior canal lateral lines and pore structure. (*b*) Ventral view CT scans showing anterior canal lateral line and pore structure. (*c*) Expanded lateral view of the preopercular and otic canals with pores marked by dotted red circles. (*d*) Expanded lateral view of infraorbital canal with pores marked by dotted red circles. (*e*) Expanded ventral view of preopercular canal with pores marked by dotted red circles. (*f*) Expanded ventral view of the mandibular canal with pores marked by dotted red circles. (*g*) DASPEI-stained superficial and canal neuromasts in the anterior region, with canals marked by dotted white lines; lateral view. (*h*) DASPEI-stained superficial and canal neuromasts in the posterior region, with canals marked by dotted white lines; lateral view. (*i*) DASPEI-stained neuromasts in the tail region; lateral view. (*j*) DASPEI-stained canal and superficial neuromasts in the anterior region; ventral view. (*k*) Expanded view of DASPEI-stained canal and superficial neuromasts in a typical section of posterior canal. IO, infraorbital; MD, mandibular; OT, otic; PR, preopercular; NA, naris; SO, supraorbtial; PO, postotic; ST, supratemporal; ATC, anterior trunk canal.

The lateral line system, sometimes described as a ‘touch-at-a-distance’ sense [1], is used by fishes to detect changes in water flow and pressure, capturing information about their surroundings. It comprises mechanoreceptors (neuromasts) that are either within subdermal channels (canal neuromasts; figure 1) or on the surface of the skin (superficial neuromasts; figure 1) [2,3]. Superficial neuromasts can be considered velocity detectors, used to detect the direction and speed of flow, while the canal neuromasts can be considered pressure gradient detectors, sensing differences in the water movement between adjacent canal pores, primarily caused by turbulence [4,5]. The lateral line system as a whole is distributed over the head, trunk and tail of the fish (figure 1), and species can differ considerably in the size and position of lateral line components [4,6].

The lateral line system plays an important role in a number of behaviours, including prey detection [6,7], alignment to flow (rheotaxis) [8–12], mate selection [13,14], conspecific aggression [13,15] and shoaling [16,17]. It has particular importance in situations where other senses are limited, for example by enabling detection of prey in dark [7,18,19] or turbid [20,21] conditions, or detecting prey buried within substrate [22]. Fishes are able to use canal neuromasts to sense vibrating obstacles [23,24] and neighbouring fish, and are able to use the lateral line as a whole to shoal [16,17]. When the sensory input from the lateral line is interrupted through surgical methods or chemical treatment, a number of behaviours are altered or lost, including normal shoaling ability [16,17,25], foraging behaviour [18,19,22], competitor assessment [13,15] and mate selection [13,14].

Typically, studies of lateral line function have compared control fish with those in which the lateral line function has been chemically or physically impaired. While these treatments are effective at inactivating the lateral line (e.g. [26]), they can cause side effects. Aminoglycoside antibiotics and cobalt chloride damage auditory hair cells [27,28] and olfactory receptor neurons [29,30], respectively. Cobalt chloride is also toxic to fish even at low exposure [29,30] and even what is considered a safe dose for lateral line ablation has a 15% mortality rate in zebrafish [31]. Additionally, some studies have claimed full or partial lateral line deactivation [16,17,26], where this may not have been true [16,17,27,28]. Lateral line transection is another method sometimes used for ablation [15,32], but this requires invasive surgery and the effects of this on fish health have not been studied. Thus, based on previous work, it has been difficult to confidently determine the exact function of the lateral line system or its various components, in relation to one another or other sensory systems.

**Figure 2.**
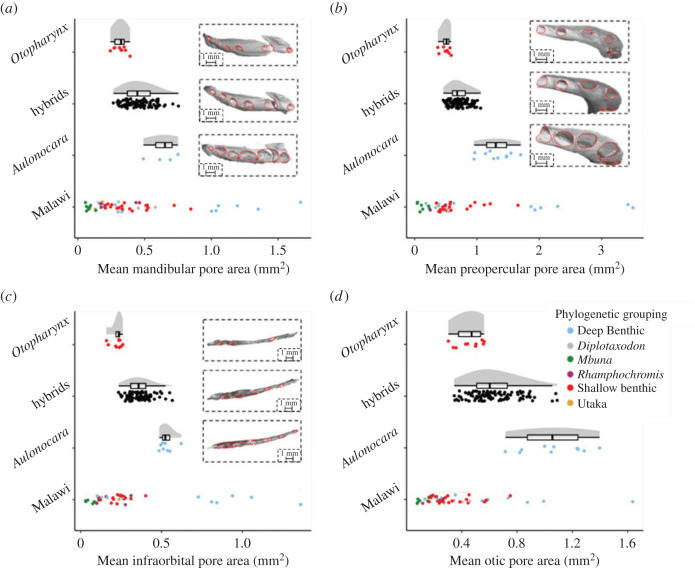
Comparative morphology of the 116 study individuals (96 hybrids, 10 *Aulonocara stuartgranti*, and 10 *Otopharynx lithobates*) (*a*) mean mandibular pore areas. (*b*) mean preopercular pore areas. (*c*) mean infraorbital pore areas. (*d*) mean otic pore areas. Mean pore areas of our study individuals are shown in contrast to a mean pore area value for 53 other species of Lake Malawi cichlids (species listed in [33]), from six key phylogenetic groups indicated by colours (electronic supplementary material, table S3). Data points in the top three categories do not correspond to the legend, and are instead labelled on the y axis. The data for the 53 other species have been taken from a study by Edgley & Genner that analysed the adaptive diversification of the cichlid family [33]. As can be seen, across all pores the *Aulonocara* has greater area. Jitter is added to illustrate the density of data points. Additional images of representative CT scans of the different regions of the canal system are also included, with each set of scans corresponding to the graph they are located in.

Here, we study associations between morphological and behavioural variation across second-generation hybrids of two phenotypically divergent Lake Malawi cichlid fishes, *Aulonocara stuartgranti* and *Otopharynx lithobates* [33] (figure 2). These species are part of the Lake Malawi cichlid adaptive radiation, in which hundreds of ecomorphologically divergent species have evolved from common ancestry in the last 1 million years [34,35]. These species possess extensive interspecific variation in lateral line systems [33,36]. *Aulonocara stuartgranti* is widely recognized as a representative of species with a ‘wide’ canal morphology (figure 2) [18,19,22,37], while *Otopharynx lithobates* is typical of species with a ‘narrow’ canal morphology, which are more commonplace in the Lake Malawi radiation (figure 2) [37,38]. Given that the expanded anterior canal pores have been associated with improved ability to catch prey in front [32], above [39,40] and even hidden in substrate [18,19,22], with the latter being seen specifically in cichlids, and that diet seems to play the biggest role in lateral line variation between cichlid species [33], it may be expected that they do not play such a role in shoaling. Equally, since superficial neuromasts in several regions of the anterior lateral line system have been associated with shoaling tendencies [41], we hypothesized that superficial neuromasts on the head and body may take a more significant role in collective behaviour in cichlids. In this study, we use the term collective behaviour to refer to shoaling or schooling behaviour where reliable alignment information is lacking.

**Figure 3.**
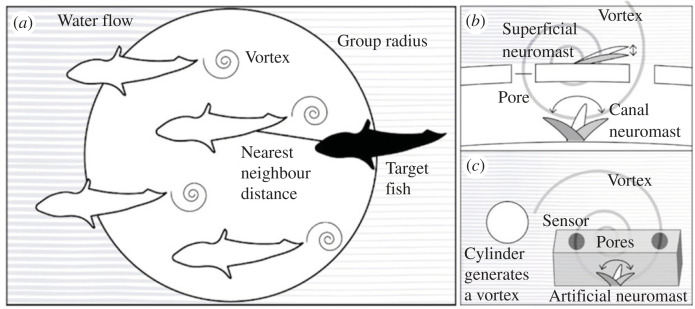
Schematic diagram summarizing different study elements: (*a*) Group of fish shedding vortices with response variables used in the experiment marked (nearest neighbour distance and group radius). Parallel lines are used to indicate background laminar flow. (*b*) Response of superficial and canal neuromasts to a vortex when in presence of background flow. The superficial neuromast is more affected by background flow, whereas the canal neuromast is less affected. The canal structure filters out background laminar flow (pale grey lines) while allowing turbulent flows like shed vortices to be detected more clearly. A stiffer superficial neuromast would be less affected by background flow, but this then has the negative side effect of causing smaller deflections in response to the important flow features, such as shed vortices. (*c*) A cylinder can be used to generate vortices (approximate to those generated by an upstream fish, albeit in different positions), and an artificial canal structure containing an artificial neuromast can detect them.

We also present the results of a hydrodynamic modelling study, investigating neuromast responses under a variety of different conditions. We specifically explored the concept that superficial neuromasts reach a state of saturation, or maximum deflection, due to background flow, which could prevent them from detecting changes in the flow, whereas the structure of the lateral line canals allows them to detect these otherwise masked flow changes, like those caused by vortices shed from upstream companions (figure 3) [1,4,5]. We also simulate a novel design of artificial lateral line and demonstrate its ability to detect the vortices shed by an upstream cylinder as an approximation of the wake of a swimming fish (figure 3) [42,43].

Overall, this study aims to investigate how differences in lateral line morphology, particularly differences between the lateral line subsystems, cause changes to collective behaviour, with sensory ability being the presumed driver. Observations about how sensing ability appears to affect collective behaviour are then explored in simulation to verify predictions. The work sheds new light on the role of the lateral line in collective behaviour of fishes and takes steps towards a novel artificial lateral line design.

## 2. Material and methods

### 2.1. Study fish

In total, we studied 116 focal fish: 10 *Aulonocara stuartgranti*, 10 *Otopharynx lithobates* and 96 *Aulonocara stuartgranti* × *Otopharynx lithobates* second generation (F2) hybrids (electronic supplementary material, table S1). Our hybrids had a mean total length of 117.31 mm (s.d. = 17.71), with the *Aulonocara* measuring a mean total length of 110.50 mm (s.d. = 10.28), and the *Otopharynx* measuring a mean total length of 117.10 (s.d. = 13.41). The second-generation *Aulonocara stuartgranti* × *Otopharynx lithobates* hybrids exhibit substantial variation in the lateral line between the ‘wide’ and ‘narrow’ canal morphologies of their parents, and collectively these hybrids span much of the range of lateral line characteristics seen across the Lake Malawi radiation (figure 2). Morphology data of the Lake Malawi radiation (figure 2) are used to frame the results of this study, giving better context and demonstrating that the two parent species are good examples of the ‘wide’ and ‘narrow’ morphologies; see Edgley & Genner for more in-depth analysis [33]. The use of F2 hybrids allows for the decoupling of genetically unlinked lateral line traits, as well as other sensory traits that enable the perception of environmental cues. Both parent species, along with many other species of cichlid, including our companion species *Hemitilapia oxyrhynchus* (a very commonplace species in Lake Malawi) shoal in natural populations [37,44–50], making them a useful candidate for these collective behaviour experiments (figure 3). Behavioural analysis showed that these companion fish tended to group tightly during experiments (electronic supplementary material, figure S4). While cichlids in Lake Malawi are not usually exposed to constant flows, the low velocity flows here enable us to more reliably encourage the swimming motions, and the wakes that they produce, that we intended to measure; analysis of no-flow conditions also showed an increased tendency to remain further apart (electronic supplementary material, figure S4). Cichlids were bred at the University of Hull. A total of 10 individuals from each of the parent species were housed separately in 90 l tanks and approximately 100 hybrid individuals were housed in a 720 l tank. A further 90 l tank held approximately 30 individuals of the species used as companion fish, *Hemitilapia oxyrhynchus*. All tanks were kept on a recirculating water system at 23°C and a 12–12 light–dark cycle. They were fed a varied diet, consisting of ZM large granular pellets, tetra tropical flake, frozen blood worm, frozen prawn, frozen brine shrimp (+ brine shrimp with supplements such as spirulina, garlic, aloe), vegetarian diet blister packs, cichlid diet blister packs, mysis, krill, daphnia, white mosquito larvae and tubifex.

### 2.2. Behavioural data

In this study, we attempt to quantify collective behaviour; this term is used over shoaling or schooling as reliable data on the orientation of individuals to calculate variables such as polarization were lacking. To do this, each focal fish was filmed in an experimental flow tank, in a group with four companion fish, in both laminar and turbulent flows (electronic supplementary material, figure S1). The experimental tank consisted of an arena of 2 × 1 m, within which foamed PVC walls were added to mimic a convergent nozzle to help control flow (electronic supplementary material, figure S2). A baffle was added upstream to give the flow time to laminarize, with additional meshes (6 mm square holes separated by 3 mm walls) added for the same purpose. The experimental area measured 121 × 55.78 cm with a water depth of 20 cm. For the turbulent treatment, turbulence was generated by vertical, clear, plastic rods of diameter 32 mm attached to the downstream side of the flow straightener. Comparisons of the resultant flows can be seen in electronic supplementary material, figure S3. The experimental tank was white and brightly lit to give fish ideal visual conditions so that any differences in visual ability would be less pronounced and be less likely to affect behaviour. Behavioural experiments took place between May and August 2018. Each trial consisted of one individual of the hybrid or parent species (the focal individual) and four individuals of the companion species. Four individuals of the companion species were used in each trial to reduce variation between trials based on morphological and behavioural differences between individuals of the companion species. With a decreasing number of companion individuals, differences in shoaling behaviour between trials would be increasingly affected by variation between the companion fish, rather than variation between the focal individuals. By varying the morphology of only one fish (our focal individual) and keeping the traits of the other group members approximately constant, variation not accounted for by the morphology of the focal individual is reduced. The five fish were netted from their stock tanks into the experimental tank and given 10 min to acclimatize with no flow. As the morphological measurements were conducted after the behavioural experiments, the fish were caught blind regarding the morphology of the individuals. Trials were then run for 20 min at an average flow speed of 7 cm s^−1^. This was calculated by measuring the distance that particles flowed downstream at several positions in the tank and then taking an average. Water flow rate was intended to be approximately 10 cm s^−1^, which was considered to be enough to induce rheotactic behaviours and encourage active swimming, while not being so high as to be a flow rate unseen in their natural environment or being overly tiring for the fish [36–38,50], but limitations of the flow tank meant that 7 cm s^−1^ was the maximum flow speed that was safely and reliably achievable. A 20 min period was deemed to be long enough to gain enough data to accurately assess shoaling behaviour and for the fish to habituate to the initially novel tank conditions so that they would express natural shoaling behaviours. After this time period, the flow was switched off again and the fish were given a further 10 min rest period. During these 10 min, the turbulence generators were either removed or inserted, depending on which treatment (turbulent or laminar) was given first. The flow was then switched back on for the second 20 min trial. Each test shoal was thus tested in both turbulent and laminar treatments, with half experiencing laminar first and half experiencing turbulent first (randomly assigned); the same companion fish were used in both the laminar and turbulent trial for a given target individual. The overall pool size of the companion fish was kept quite small to lessen the effects that variation in this population might have on the collective behaviour metrics. As each companion individual was not to be tested more than once per day to prevent overly tiring or stressing any individuals, and assuming that seven trials could be completed in a day, a minimum of 28 companion fish were needed. It was expected that the large number of trials and the constant mixing of companion individuals would average out any effects caused by differing companions over the course of the experiment. Treatment order was also divided such that half of the individuals with blue coloration, assumed to be breeding males, experienced laminar first, with the same approach used for the plain-coloured individuals, assumed to be females or juveniles. After the testing, the focal individual (i.e. hybrid or parent) was placed in isolation in a smaller 45 l tank to await further steps of the experiment, while the companion individuals were kept separate for the remainder of the day to prevent them being tested again.

### 2.3. Recording conditions and video analysis

Recordings were made using a Panasonic VX870 camera, filming in 4K at 25 fps. The camera was suspended at a height of 148 cm above the base of the experimental tank. The camera was zoomed so that the experimental arena filled the entirety of the screen. The camera filmed in 9 min segments, and these were stitched together to form a single 20 min video per trial, and quality was reduced to 1080p, all using ShotCut (https://shotcut.org/). The automated video tracking software idTracker v. 2.1 [51] was used to extract X-Y positional coordinates. These were analysed in Matlab R2013b. Mean nearest neighbour was calculated by determining the distance between all individuals in every frame, then taking the lowest of these values for the focal individual; the mean across all frames was calculated from this. Group radius was calculated by finding the central point of the shoal (the mean of all of the individual's coordinate points) in each frame then calculating the distance to the individual furthest from the centroid. This was highly correlated to an alternative measure of group cohesion, the bounding hull circumference (electronic supplementary material, figure S4). The focal fish's distance to the group centroid was also calculated and also found to be highly correlated with the group radius (electronic supplementary material, figure S4). Nearest upstream neighbour and nearest ‘field of flow sensing’ neighbour metrics were calculated from the nearest neighbour distances. Neighbours were deemed upstream if they had an X coordinate less than that of the target individual, while those deemed in the ‘field of flow sensing’ had both a lower X coordinate and had Y coordinate within 5 cm of the target individual. The origin of the coordinate system was at the most upstream and furthest left point of the experimental tank (the bottom left corner in the images used during analysis), and the environment was oriented such the X axis pointed downstream, and the Y axis pointed cross-stream. Values where neighbours were not in front or not directly in front respectively were excluded. These metrics were included because it is not possible to detect downstream individuals using the lateral line, as the hydrodynamic stimuli generated by tail beats cannot travel upstream. In this way, we attempt to better ensure that it is lateral line input causing the observed behaviour. Each video was watched manually in idPlayer [51] to verify that the identity of the focal fish remained the same within the two 20 min trials.

### 2.4. DASPEI staining and imaging

After three focal fish were tested, which was usually within approximately 3 hours, but occasionally individuals were left overnight before this next step, focal individuals were transferred to a DASPEI solution of concentration 0.01 mg ml^−1^ where they were left for 30 min. They were then euthanized by submersion in a 300 mg l^−1^ solution of Tricaine methanesulfonate (MS-222) for 10 min, followed by destruction of the brain with a sharp implement. Each individual was then transferred to a platform placed 36.3 cm underneath the Sigma 18–200 mm f/3.5–6.3 lens on a Canon 550D DSLR camera; the manual zoom was adjusted so that the fish occupied the entire screen. The camera was connected to a laptop and controlled by the Canon EOS Utility Software, allowing all adjustments to zoom and focus to be made from the laptop. A Royal Blue lamp (465 nm) was directed at the fish to excite the DASPEI stain. A yellow glass filter (long-pass filter, 500 nm) was placed in front of the camera lens to remove interference from the excitation light to allow the emission light to be seen more clearly. Multiple stills were taken of each individual at a resolution of 5184 × 3456 with the lamp redirected to highlight different areas of the fish to ensure that all DASPEI labels were visible; multiple focal depths were used for each light position too. These images were then compiled using the ImageJ (https://imagej.nih.gov/ij/) stack feature to generate a single image showing all areas of the fish highlighted. The result was a lateral view of the whole fish. Images captured in this way gave sufficient detail to count the canal and superficial neuromasts visible at the surface, henceforth referred to as ‘visible superficial neuromasts' or ‘visible canal neuromasts’. From this, we quantified six neuromast count variables: anterior superficial, posterior superficial, posterior canal, lower posterior superficial, lower posterior canal and tail neuromasts. After neuromast imaging, individuals were ethanol preserved.

### 2.5. Computed tomography scanning and morphometrics

All individuals were scanned using a Nikon XTH225ST Computed Tomography (henceforth referred to as CT scans) system in January 2019. We scanned fish two at a time, each scan using 3141 projections and a voxel size of 20–30 µm. CT scan parameters were determined following preliminary scans of similar specimens, and careful inspection of the cranial pores following reconstruction. Stacks of images were imported into VG Studio 3.0 (Volume Graphics GmbH) and reconstructed into a three-dimensional model. From these three-dimensional reconstructions, we captured two-dimensional images from the ventral head perspective and lateral head perspective (on the left side of the fish). From these images, four anterior lateral line canal pore size variables were quantified: mean otic canal pore size, mean preopercular canal pore size, mean dentary canal pore size and mean infraorbital canal pore size (figure 1).

To quantify differences in cranial lateral line canal pore morphology, we used a landmark-based morphometric approach. Using the two-dimensional images generated from CT scanning, we digitized landmarks and semi-landmarks to capture morphological variation using tpsDig 2.30 for Windows [52]. We drew a curve around the circumference of each pore, beginning and ending at a homologous anchor point (the anterior limit of the lateral line canal pore). These curves were resampled resulting in 10 equidistant semi-landmarks, which were subsequently converted to landmarks in tpsUtil64 1.74 for analysis.

For the images showing the ventral side of the head (figure 1), we collected landmarks for pores 2–5 of the mandibular canal (figure 1) and pores 1–4 of the preopercular canal (figure 1). From lateral head images (figure 1), we collected landmarks for pores 5–7 of the preopercular canal (figure 1) and the two pores of the otic canal (figure 1). We also measured head size for each specimen, defined as the distance from the anterior limit of the dentary bone to the posterior limit of the operculum. All landmarks were digitized by the same individual, and with only short breaks between digitizing sessions to ensure consistency. We tested for digitization error by subjecting 20 of our individuals to repeated landmarking. Using analysis of variance, we tested for significant differences in mean dentary canal pore area, among both individual and landmarking events (repeat or original landmarking). We found no significant difference between landmarking events (original or repeat) (*F*_1,19 =_ 0.3655, *p* = 0.5526), but highly significant differences between individual specimens (*F*_19,19_ = 159.77, *p* < 0.001). We visualized this difference by performing a Procrustes fit on landmark data using R 3.6.1 [53], the package geomorph [54] and a principal component analysis using Procrustes coordinates (electronic supplementary material, figure S4).

Following conversion to landmarks, for each pore, we discounted the 10th landmark (as its coordinates were the same as the first in each instance). We used the remaining nine landmarks' coordinates as vertices to create a polygon, and calculated the area of this polygon using the polyarea() function in the geometry package [55]. These areas were used as approximations for pore area for each specimen. Despite these being slight underestimates of the true pore area, our methodology was consistent across all individuals.

### 2.6. Statistical analyses

R v. 3.6.1 [53] was used for the statistical analyses. Generalized linear mixed models (GLMMs) with negative binomial distributions were developed using the glmmadmb package [56,57]. These models are used to establish a relationship between a response variable and a predictor variable, where a number of other explanatory variables can be included in the models to account for other effects. The response variable in our models was the mean nearest neighbour distance, the mean upstream nearest neighbour distance, the mean ‘field of flow detection’ nearest neighbour distance or the mean shoal radius. The primary predictor variable of interest was the focal lateral line variable, while the species (*Aulonocara stuartgranti*, *Otopharynx lithobates* or hybrid), focal fish's body length, treatment (laminar or turbulent) and whether it was the first or second trial for this focal fish were explanatory variables. The ID of the fish was included as the random factor throughout as each focal fish was tested in both laminar and turbulent flow.

To determine which lateral line explanatory variables explained the most variation in the behavioural response variables, we used the corrected Akaike information criterion (AICc). Specifically, comparisons between AICc value for each model were made using the ICtab function in the bbmle package [58]. Comparing models in this way does not give absolute information about each model's predictive power, but a relative comparison of likelihood between models. We thus included in the model comparisons a null model: a model that relates the response variable to all of the explanatory variables, but excludes the primary predictor variable, which was a lateral line variable; the null model still includes body length. Models with lower AICc values have higher support and are more likely given the data. We considered a model with an AICc of two or more units lower than the null model (i.e. ΔAICc ≥ 2) to have strong support. By comparing AICc values in this way, we determined which aspects of our focal fish's sensory morphology best predict the shoaling metrics. The method of comparing GLMMs using the AICc was chosen because mixed effects models are useful when comparing data with more than one source of random variability, in this case, as a result of the repeated measures of position data (of five different individuals) over time.

### 2.7. Sensor simulations

A computer-aided design (CAD) model of a cylinder measuring and an artificial neuromast sensor was designed using Autodesk Fusion v. 2.0.8816. Cylinder diameter was chosen to be 100 mm, approximately equal to the mean fish body length. The artificial lateral line sensor was designed to mimic a limited section of canal, being cuboid in shape and measuring 25 × 25 × 50 mm, with a highly elastic haircell acting as the neuromast; this is deliberately macro-scale for twofold reasons. Firstly, to determine if the behaviour of the canal section at this size can be considered similar to the biologically accurate size, and to improve the ease with which it can be mass produced. Two evenly spaced, identical holes were cut out of one face to act as pores. Five different pore diameters were used to investigate the effect that this would have on the neuromast response to external flow stimuli. This was determined by measuring the mean and the range of the flow velocities within the sensor, which arise due to external pressure differentials across the pores. A larger range of velocities indicates a greater, so more easily measured, response to a stimulus; the stimulus is the same for each sensor. Rising mean velocity indicates a reduction of filtering effect. Flow speed is also measured in the absence of a canal structure. The sensor was centred on the origin, with the cylinder placed 200 mm away in the upstream direction and 40 mm to one side. stl files were generated from these models. OpenFOAM (blueCFD-Core 2017) was used to simulate flow speeds of 0.5 m s^−1^ over 400 s. Snappyhexmesh was used to convert the stl files to be used in the OpenFOAM simulation [59]. Results were exported as Excel files containing the velocity data from every point in the mesh. Matlab 2018b and R v. 3.6.1 [53] were used to determine the point of maximum variation, and to generate graphs and raincloud plots of flow velocities. Velocity data at the position of the artificial neuromast hair cell were also extracted from OpenFOAM using the Plot Over Line function. This function takes the data from all the mesh points along a desired one-dimensional line.

## 3. Results

### 3.1. Lateral line morphology and behaviour of focal fishes

Across the full dataset, lateral line morphology and body size were strongly correlated, with larger fishes having increased numbers of visible superficial neuromasts and larger cranial canal pores (electronic supplementary material, figure S5). After correcting for fish total length, individuals of *A. stuartgranti* were characterized by larger cranial canal pores than *O. lithobates*, but fewer visible anterior superficial neuromasts (figure 2; electronic supplementary material, table S1). Hybrids possessed a broad range of phenotypes, encompassing and spanning the two parental phenotypes (figure 2; electronic supplementary material, figure S6–S8, table S1).

Mean nearest neighbour distance and mean shoal radius of the hybrids was intermediate between the two parental species (electronic supplementary material, table S1). This pattern persisted when considering only those periods of time when nearest neighbours were either immediately upstream or within the 'flow detection field' of the focal fish (defined as a channel in front of the fish with boundaries at 50 mm either side; electronic supplementary material, table S1).

### 3.2. Associations between lateral line morphology and behaviour

**Figure 4.**
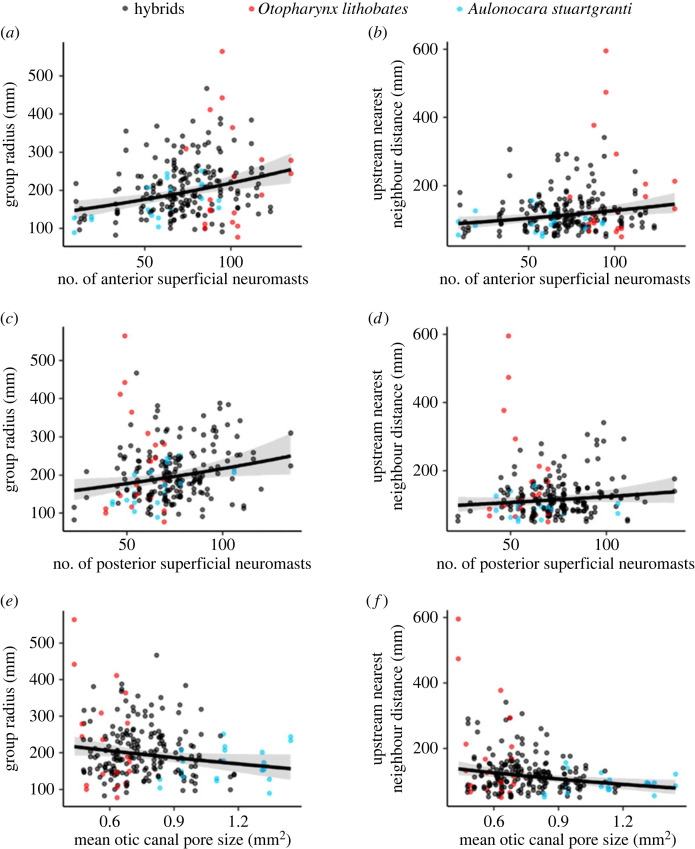
Generalized linear mixed models (GLMMs) of associations between lateral line morphology and behaviour. In each graph, there are 232 points, with two points for each fish, one in the turbulent treatment and one in the laminar. Black points indicate hybrid individuals, red points indicate the individual was an *Otopharynx,* and blue points indicate the individual was an *Aulonocara.* (*a*) Number of anterior superficial neuromasts and group radius. (*b*) Number of anterior superficial neuromasts and distance to nearest upstream neighbour. (*c*) Number of posterior superficial neuromasts and group radius. (*d*) Number of posterior superficial neuromasts and distance to nearest upstream neighbour. (*e*) Average areas of preopercular canal pores and group radius. (*f*) Average areas of preopercular canal pores and distance to nearest upstream neighbour. Each graph here shows a significant relationship for the complete dataset, with the exception of the posterior superficial neuromasts affecting upstream nearest neighbour distance; this was only significant when affecting the hybrids only. Significant relationships are those identified in the AIC comparison process where a model is 2 or more fewer than the null model, which represents a random relationship.

Increased numbers of anterior and posterior superficial neuromasts were positively related to the group radii (figure 4; table 1). More anterior superficial neuromasts increased the distance to nearest upstream neighbours and neighbours in ‘field of flow detection’ for the complete dataset, while more posterior superficial neuromasts also increased these distances in the hybrids-only dataset.

**Table 1.** Summary of the models with strong support (ΔAICc ≥ 2). ‘+’ indicates a positive slope between the behavioural and morphological variables (green shading), ‘−’ indicates a negative slope (blue shading), U indicates an unsupported result, * indicates that there is also a strongly supported interaction between the morphological variable and the treatment (laminar/turbulent flow).

morphological variable	mean nearest neighbour distance		mean group radius		mean nearest upstream neighbour distance		mean nearest neighbour distance within ‘field of flow detection’	
	all	hybrids	all	hybrids	all	hybrids	all	hybrids
anterior superficial neuromasts	U	U	+*	+	+*	U	+*	U
posterior canal neuromasts	U	U	U	U	U	U	U	U
posterior superficial neuromasts	U	U	U	U	U	+	U	+
lower posterior canal neuromasts	U	U	U	U	U	U	U	U
lower posterior superficial neuromasts	U	U	U	U	U	U	U	U
tail neuromasts	U	U	U	U	U	U	U	U
otic canal pore size	—*	U	U	U	—*	U	—*	U
preopercular canal pore size	—	U	—	U	—*	U	—*	U
dentary canal pore size	—	U	U	U	—	U	—*	U
infraorbital canal pore size	—*	U	U	U	—	U	—*	U

It was identified that there was an interaction with the flow regime for each of anterior superficial neuromast models, such that fish with increased numbers of these neuromast tended to have larger group radii in turbulent flow than in laminar, and tended to be further from upstream neighbours and neighbours in the ‘field of flow detection’. For the hybrids-only dataset, individuals with more posterior superficial neuromasts formed tighter groups in turbulent regimes than in laminar for both upstream nearest neighbour distance and distance to nearest neighbour in ‘field of flow detection’. See electronic supplementary material, figure S9 for more information.

Our GLMMs revealed that all measured canal pore variables (mean otic, preopercular, dentary and orbital pore sizes) were negatively associated with nearest neighbour distances when using the complete dataset (figure 4). However, for the hybrids-only data, we found no supported associations between canal pore size and collective behaviour, suggesting the overall pattern in the complete dataset was driven by the data from the parent species.

### 3.3. Artificial sensor simulations

**Figure 5.**
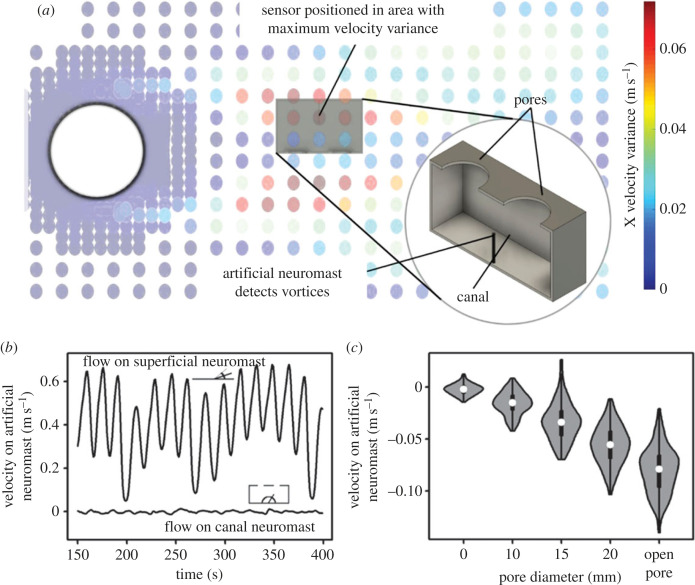
The design of the simulated artificial lateral line sensor and its location and response in the simulated environment. (*a*) Flow velocity variation within the simulated flow tank during a 400 s simulation, showing areas of high levels of flow velocity variation (red) and low levels of flow velocity variation (blue). Each coloured dot in simulation marks a mesh point. The sensor was positioned at the point with highest variation. The position and CAD model for the artificial sensor used in simulations are shown. The CAD model is inspired by the structure of a section of canal lateral line, with a single neuromast being positioned between two pores. (*b*) Comparison of flow velocities seen in flow where no structure is present (upper line) and flow velocities within the canal structure with pore size 15 mm (lower line) (shown in (*a*)). Schematic drawings of the predicted response that a neuromast of similar stiffness would show without the canal structure (like a superficial neuromast) (top left) and with it (bottom right) are included. (*c*) Distribution of the speeds seen within the artificial canal with varying sizes of pore, where wider regions of each ‘violin’ indicate more time with flow at the associated velocity, with the black bar representing the interquartile range and the white dot representing the mean. ‘Open pore’ indicates that there are no pores and instead the entire surface bearing pores (seen in (*a*)) is removed to give a single maximum-size pore. Velocities are negative as the sensor is positioned at the point where flow is reversed.

Analysis of simulated flow behind a 100 mm diameter cylinder (used to generate a wake similar to one generated by a swimming fish [42,43]) at a flow speed of 0.5 m s^−1^ (electronic supplementary material, figure S10) revealed areas with substantially higher levels of variation in flow speed (figure 5). Our computational model predicted that the location with the greatest flow speed variation was 200 mm behind the cylinder, and 40 mm offset from its centreline. Data were extracted from here, initially with no canal structure, showing the flow speeds that a superficial neuromast haircell would experience. This data gave an oscillating waveform of set period (figure 5) that is a result of the repeated changes in flow velocity that occur when flow is slowed or even reversed on one side of each successive shed vortex. The waveform oscillated around a mean flow speed of 0.4 m s^−1^, slightly slower than the background flow speed set in the simulation, which is due to the flow being slower in the vicinity behind the cylinder. Flow speed data taken from the same location, but from within the simulated canal-like sensor showed a large decrease in the mean flow speed (figure 5). This decrease shows the filtering effect that the sensor has on background flow speed, allowing it to measure only the velocity that is induced as a result of the pressure differential between the pores. In figure 5, it can be seen that the mean value of the flow velocity in the sensor is close to 0. Such low velocity might be thought to be below the threshold needed to illicit a response from the visual tracker in a physical set-up. However, additional preliminary trials were done with a physical three-dimensional printed sensor, where it was demonstrated that the vortex created by a passing cylinder was able to be detected by a sensor with 10 mm pores and larger (electronic supplementary material, figure S11).

Our simulations of how a range of pore sizes impacted the sensitivity of the sensor showed that as the diameter of the pore increased, so did the flow velocity acting on the artificial neuromast (figure 5). There was also a trend of increasing variability in velocities experienced by the neuromast, although these velocities were still well below what the superficial neuromast could be expected to experience (figure 5). Given that the responses for all of the different pore sizes are to the same stimuli, i.e. the wake behind a 100 mm cylinder, this shows that a larger pore size results in a greater response. In turn, it can be said that as the strength of the stimuli decreases, the response from each pore size will lessen accordingly, with the response from the 5 mm pores becoming undetectable first, then 10 mm, etc. In this way, a larger pore size is shown to be better able to detect upstream stimuli, like the vortices from an upstream fish. The results here were echoed in the preliminary trials with the three-dimensional printed physical sensor, where 5 mm pores showed no response to the passing vortex, but 20 mm pores showed a strong response (electronic supplementary material, figure S11).

## 4. Discussion

Together, the hybrids exhibited a broad range of morphological and behavioural diversity between the two parental phenotypes of ‘wide’ canals and ‘narrow’ canals, with most traits, on average, being of intermediate values. These results provide evidence of the heritability of several lateral line components, such as pore size and neuromast number [60]. Similarly, the collective behaviour of the hybrids, as measured by different metrics of group cohesion, was intermediate between the two parental phenotypes, contributing additional evidence to the existing literature that shoaling in fish has a heritable component [41,61,62]. Using F2 hybrids enabled us to partially overcome difficulties of testing two distinct species, where there is the risk that differences in behaviour arise from distinct species-specific behaviours or information from other sensory systems, such as olfactory or visual abilities. Given the known roles of these senses, particularly vision, in shoaling and schooling behaviours, it is important that efforts are made to control them such that any variation in behaviour can be better attributed to the lateral line. Since these two fish live in similar conditions, close to the seabed at a depth of around 10 m, with the primary difference being that the *Aulonocara* lives over sandy substrate while the *Otopharynx* lives over rocky substrate [37], we make the assumption that they possess similarly developed vision. While we acknowledge the important role of vision in shoaling, we assume that all hybrids possess similar levels of visual acuity and we ensure perfect visual conditions, so we can attribute any changes in observed behaviour to variation in the lateral line.

Although vision is likely to be the main sensory modality used to mediate collective behaviour in many fish species [17,21,63–65], our results provide further evidence that information from the lateral line system contributes to collective behaviour. However, while it has long been known that the lateral line system is important in informing collective behaviour in fish [16,17,25], and that superficial and canal neuromasts differ in the sensitivity to different water movement characteristics [1,4], the specific roles of the different regions of lateral line system were yet to be established. Larger anterior canal pores were associated with a decreased average proximity to nearest neighbours (figure 4) and a decreased group radius. Larger pores offer the canal system greater exposure to external flow conditions, giving a fish more information about its surroundings and the potential for better control responses, while flow around anterior structures is less disrupted by the body of the fish, boundary layer effects and self-generated flows, particularly from the pectoral fins [66], making incoming information more accurate. This is supported by evidence that the undulatory swimming motions of fish are optimized to reduce self-generated pressures around the head [67]. Additionally, fish can respond to a stimulus more quickly, as rheotactic behaviour will expose the head to upstream stimuli first. Studies have shown that fish gain many benefits from shoaling in tighter formations [68–71] and as such, improved control and acuity can aid this.

Fish with more visible anterior and/or posterior superficial neuromasts tend to form less cohesive groups. As superficial neuromasts detect flow velocities [1,4], they could give broad warning of obstructions (like companions) ahead that have reduced flow velocity behind them. More superficial neuromasts may make individuals more sensitive to these reduced velocity areas and naturally distance themselves from them, resulting in more widely spaced fish. Increased numbers of superficial neuromasts also increase susceptibility to noise, resulting in a greater margin of error in estimating distances to shoal mates, and potentially then causing fish to stay further apart. The propensity to remain more distant when uncertain could be in order to mitigate possible collisions. Previous work has highlighted the importance of lateral line mechanoreception in preventing collisions within a shoal, with some lateral line-ablated fish colliding with their shoal companions with sufficient force to stun themselves [16,17,25]. Collisions can have significant negative effects for the individual, so an increased sensitivity to slower flow regions may act to avoid physical contact with shoal mates.

Shoaling and schooling are important behaviours that serve multiple purposes in large numbers of teleost fishes [42,68,72–79]. To govern the complex dynamics seen in these systems [75,76], evidence suggests that vision [72–74,77], the lateral line [16,17,25], audition [78] and olfaction [79] can be involved, making it difficult to understand the distinct roles they play. Additionally, recent work shows that genetics can play a role, with mutations in certain genes affecting shoaling behaviour in Zebrafish [61]. On the other hand, the lateral line has been shown to be essential in regulating not just shoaling [16,17,25], but also rheotaxis [8–12], conspecific aggression [13,15], mating [13,14], and prey detection [6,7,18–20,22]. These behaviours may drive lateral line adaptations to differing degrees and the relative importance of each will depend on the ecology of the individual species and populations. For example, cave-living eyeless forms of the Mexican tetra *Astyanax mexicanus* have almost entirely lost their shoaling behaviours and, despite increased numbers of neuromasts, lateral line ablation does not significantly affect this [65], suggesting their lateral line plays other roles. In firehead tetras, however, ablation of the lateral line renders an individual unable to shoal normally [25]. It has also been shown that the mandibular canal is correlated with feeding behaviour and diet in cichlids [18,19,22,33]. As such, adaptations of the lateral line may not primarily be driven by shoaling, but as we show, variation in the lateral line morphology of the individuals we tested does interact with their collective behaviour.

We hypothesized that superficial neuromasts would be the primary correlates of collective behaviour, with anterior canal pores taking a smaller role. Our results partially support both predictions, with superficial neuromasts mediating group radius, and increased numbers resulting in less cohesion. This is in line with research showing that removal of superficial neuromasts of the trunk results in the target individual becoming more cohesive with the group [16,17]. We found that the role of the anterior canal neuromasts was greater than expected, with larger canal pores in this region resulting in decreased nearest-neighbour distances. It is possible therefore that superficial neuromasts regulate repulsion from neighbours while canal neuromasts regulate attraction.

Our modelling of an artificial lateral line demonstrated that a single artificial neuromast within a section of canal was better able to filter out background flow than an artificial superficial neuromast (figure 5), highlighting the important functional differences between the two sub-systems [1,4]. Additionally, the reduced flow speeds within the sensor allow for the use of a more flexible neuromast, resulting in greater potential deflection in response to low-velocity flows within the sensor, thereby increasing sensitivity. By contrast, a more flexible neuromast in the open flow will deflect significantly from the speed of the background flow, with the potential that maximum deflection will occur, lessening or preventing responses to other stimuli, like an upstream fish, from being recorded. Previous work has shown that flow of around 10 cm s^−1^ is enough to mask the vibrations caused by a vibrating sphere in both still-water (goldfish) and riverine (rainbow trout) fish [80]. Increasing the stiffness of the superficial neuromast might prevent maximum deflection at the same flow velocity, in turn preventing saturation, but by doing so, less deflection will be recorded in response to flow stimuli, reducing the sensitivity of the artificial lateral line.

We also found that a larger pore size resulted in both an increased mean flow velocity and an increased range of flow velocities. The latter entails greater deflections in response to vortices. It also implies that the threshold velocity for the sensor to be able to detect vortices is lower. However, the increasing mean flow velocity showed there was less filtering of the background flow at larger pore size. These trends have been demonstrated before with artificial canal neuromast sensors that were kept at the expected biological scales, but we show that the trends are also present in a macro-scale sensor. Further work has been undertaken to fully optimize the sensor for use in swarm robotics applications [81]. It should be noted that the low internal flow velocity does have the potential to reduce the efficacy of the sensor, but that the future work addresses and overcomes this [81]. At this stage, the design is only intended to be a proof of concept that confirms the observations and shows that a macro-scale canal can be effective.

It should be noted that the bony pores of cichlids are covered with a thin membrane that limits flow entering the canals; only a small hole exists that fluid can pass through [82]. A larger area of membrane however can act to amplify external signals making them more perceptible within the canals [82]. Our sensor design adopted this principle but attempted to simplify it by removing the membrane, which enabled us to explore if it was still effective at detecting pressure changes. Larger pores here resulted in an increase in both the mean flow velocity and the range of velocities detected, indicating that with increasing pore size, the sensor gives a greater response to an external stimuli. The external stimuli is the same in each case, i.e. the vortex street.

Significant work has already been done in the field of artificial lateral lines (reviewed by Hu *et al.* [83]), but the majority of these systems use micro-electrical-mechanical systems (MEMS) [84–86], remaining true to the scales of the biological systems they are based on. This is because flow properties change significantly as scales do. However, MEMS can be difficult to mass produce [87]. Some artificial lateral lines that use off-the-shelf pressure or optical sensors exist [88,89] but these use multiple sensors per lateral line to effectively localize a source, increasing cost and complexity. In this work, we show that a single instance of a macro-scale sensor is able to effectively filter background flow velocity and output a sinusoidal signal (similar to the input); with further work, this could be used to help underwater swimming robots to locate and follow other swimming robots without the need for visual input. This is especially helpful in the underwater environment which is often dark or turbid. We also show that larger scale artificial canal lateral line sensors are feasible, something that has not been explored, which could open new areas of research in artificial lateral line design. Additionally, the simplicity of the proposed design makes it easy and inexpensive to mass produce (electronic supplementary material, figure S9). As such, it will be useful in the field of swarm robotics, where the numbers involved prohibit anything expensive or difficult to manufacture. Swarms of underwater swimming robots could then be used for environmental monitoring or search and rescue, where the large numbers improve area coverage and parallel processing. A number of systems exist that use an artificial lateral line to gain information about a neighbouring individual [90–93], which mark the first steps towards an artificial lateral line controlled swarm, and this work could help to reduce the cost and complexity of the lateral line required.

## 5. Concluding remarks

Our results have implications for understanding the lateral line system of fishes in the context of their evolution, ecology and behaviour. We demonstrate how to quantify the morphological disparity within multiple lateral line system structures, and to test the consequences of this variation on collective behaviour. Our results also highlight how different aspects of lateral line system morphology differentially affect shoaling behaviour, and specifically identify the importance of the head structures in shoaling. This could help to explain how globally important shoaling species like herrings, shads and sardines, that have well-developed anterior lateral line canal morphology [94,95], albeit different to cichlids, are able to coordinate their collective movement in the absence of posterior lateral line canals [94,95]. In addition, these findings can inform the design of artificial lateral lines. Biomimetic artificial lateral line systems already exist [83–86], but these are currently still complex and can be expensive to mass produce. The design and the simulation results presented here could help to develop a simple, inexpensive, macro-scale sensor capable of informing minimalistic underwater robots for swarming purposes, eventually without the need for visual signals.

## Data Availability

All data and code used for this paper are available from the Dryad Digital Repository: https://doi.org/10.5061/dryad.4xgxd257h [96]. Supplementary material is available online [97].
